# Discharge After “did Not Attend” in Working Age Adults Community Teams

**DOI:** 10.1192/bjo.2026.11739

**Published:** 2026-06-30

**Authors:** Abbigayle Buctkon-Perkins, Salah Elhaddad, Ejura Opaluwah, James Whelan

**Affiliations:** Leeds and York Partnership NHS Foundation Trust, Leeds, United Kingdom

## Abstract

**Aims:**

Recent high-profile incidents involving patients who disengaged from services and later caused serious harm have raised concerns within the psychiatry community about discharging service users after repeated non-attendance.

This prompted us to review our own discharge pathways and risk-assessment processes for patients who do not attend (DNA) their appointments.

We aimed to assess compliance within current CMHT (community mental health team) discharge procedures for patients who DNA, identify inconsistencies in practice, and develop standardised processes to ensure safe, consistent practice.

**Methods:**

We performed a retrospective audit using a sample of the 40 most recently discharged patients who had a discharge reason of “did not attend”. After initial review of the records to assess if the patient was truly discharged after DNA, 35 of those patients were then suitable for analysis.

The setting is CMHT South East and South West in LYPFT (Leeds and York partnership foundation trust) and we collected data on patients who were discharged from October 2023 to October 2024.

We collected data via analysing digital patient records, clinic and discharge letters, and minutes from multi-disciplinary team (MDT) meetings.

We produced a proforma to check the results against the trust DNA policy. We also then analysed patient data for 6 weeks after discharge to assess for adverse outcomes following discharge after DNA.

**Results:**

We found that there was a 63% overall compliance with the existing DNA policy. Patients were contacted on average 3 times, and this was documented thoroughly in 80% of contacts.

A risk assessment was documented in 53% of cases, and was felt to be “thorough” in 75% of cases where it was present.

MDT discussions were had prior to discharge in 60% of cases.

Patients were informed of their discharge in 76% of cases.

In regard to adverse outcomes in the 6 weeks following discharge, there was 1 inpatient admission, 1 patient who was seen in a 136 suite, 1 arrest, and 6 patients were re-referred back into the service.

**Conclusion:**

Overall we found that clinicians made multiple contact attempts to patients before discharge, and often involved the MDT. However, there was poor documentation of risk assessments, and safeguarding considerations were rarely documented.

We recommended posters in clinical areas to refresh clinicians on the DNA policy and produced a proforma for clinicians to use to document their risk assessment and decision making when discharging a patient after DNA. We plan to re-audit after these interventions to assess impact.

